# Incidence and predictors of urinary incontinence rates after thulium fiber laser enucleation of prostate performed by single surgeon

**DOI:** 10.1080/20905998.2024.2330737

**Published:** 2024-03-22

**Authors:** Armais Albertovich Kamalov, Nikolay Ivanovich Sorokin, Andrey Alekseevich Strigunov, Olga Yurevna Nesterova, Ilya Vladimirovich Bondar

**Affiliations:** a Academy of the RAS, Medical Research and Educational Centre, Lomonosov Moscow State University, Moscow, Russia; b Department of Urology and Andrology, Faculty of Fundamental Medicine, Lomonosov Moscow State University, Moscow, Russia; c Urology and Andrology Unit, Medical Research and Education Centre, Lomonosov University, Moscow, Russia; d Urologist, Medical Research and Education center, Lomonosov Moscow State University, Moscow, Russia; e Urologist, Researcher, Urology and AndrologyUnit, Medical Research and Education center, Lomonosov Moscow State University, Moscow, Russia; f Faculty of Fundamental Medicine, Lomonosov Moscow State University, Moscow, Russia

**Keywords:** Thulium fiber enucleation of benign prostate hyperplasia, thulium fiber laser, urinary incontinence

## Abstract

**Purpose:**

We aimed to evaluate the predictive clinical and operative risk factors for urinary incontinence (UI) rates following thulium fiber enucleation of benign prostate hyperplasia (ThuFLEP).

**Materials and methods:**

We conducted a retrospective cohort review of a prospectively maintained database for patients who underwent ThuFLEP between 2021 and 2023 in our university clinic performed by a single surgeon with high experience (more than 3000 endoscopic enucleation). To perform the operation, we used a thulium fiber laser Fiberlase U3 (IRE Polus ltd, Fryazino, Russia) with operating modes of 1.5 J and 40 Hz 60 W. Postoperative UI rates and risk factors following ThuFLEP were assessed for three follow-up intervals (up to 1 months, from 1 to 3 months and after 3 months post-ThuFLEP). Statistical analysis was conducted using univariate and multivariate logistic regression.

**Results:**

Out of total 578 patients who underwent ThuFLEP, the study included 381 patients with full information about continence status. Long-term UI (more than 3 months) were found only in 1.6% cases with 0.8% for stress UI, 0.3% for urge UI and 0.5% for mixed UI. Univariate logistic regression analysis has shown that higher total laser energy (OR = 1.009; 95% CI = 1.001–1.018; *p* = 0.036) and enucleation technique (OR = 0.272; 95% CI = 0.113–0.656; *p* = 0.004 in favor of En-bloc ThuFLEP) were associated with UI overall, while at multivariable analysis only En-bloc ThuFLEP was protective factor for UI (OR = 0.302; 95%CI = 0.123–0.739; *p* = 0.009). For short-term UI (less than 1 months) early sphincter release was found as protective factor at both uni- and multivariate analysis compare to longer UI.

**Conclusion:**

Short-term UI up to 3 months following ThuFLEP is associated with early sphincter release, while overall UI rates are related with enucleation technique (preferable En-bloc) and total laser energy (preferable lower energy). The surgeon should take all these risk factors into account before choosing the technique of BPH surgical treatment.

## Introduction

For several decades, transurethral resection of the prostate (TURP) has been considered as a gold standard procedure for the treatment of benign prostate hyperplasia (BPH), occurring in more than 40% of men over 60 years of age [1]. However, the occurrence of endoscopic enucleation has triggered a paradigm shift in the surgical treatment of BPH. Surgical management of BPH is ever evolving with multiple different approaches, including endoscopic enucleation, that has been increasingly utilized as one of the gold standard treatments for BPH in recent decades.

Endoscopic enucleation for BPH can be performed with bipolar or laser energy: Tm:YAG laser (ThuLEP), Ho:YAG laser (HoLEP), diode laser, potassium-Titanyl-Phosphate (KTP) and the lithium triborate (LBO) laser. In 2016 Thomas R. W. Herrmann conclude that all enucleation equipment techniques are similar and can be summarized under the term endoscopic enucleation of the prostate [2]. However, it was before newly invented thulium fiber laser (Tm-fiber) was introduced in clinical practice.

Despite the proximity of the technology, the Tm-fiber differs in its characteristics having a different wavelength (1.94), which corresponds to the peak of water absorption. Also, the Tm-fiber, unlike the Tm:YAG laser, has a pulsed mode of operation, like a Ho:YAG laser [3]. Thus, Tm-fiber combines all the best advantages of the thulium and holmium laser and today it is believed, that Tm-fiber is one of the best lasers for the treatment of BPH.

According to previous studies Tm-fiber enucleation (ThuFLEP) has shown high effectiveness, long-term symptoms relief, low complication and high patient’s satisfaction rates [4]. However, one of the main potential adverse outcomes following endoscopic enucleation including ThuFLEP is the development of de novo urinary incontinence (UI), that is common, but typically transient [5]. Reported postoperative incontinence rate is different from different studies with great heterogeneity. According to meta-analysis, published in 2022 there was no significant difference in incidence of short-, intermediate-, and long-term UI for various prostate enucleation procedures. However, it was only one article in meta-analysis with ThuFLEP, that was assessed together with thulium:YAG laser [6].

Possible risk factors for developing UI after different types of endoscopic enucleation include age, increased prostate size, enucleation technique (en-bloc or not with early sphincter release or nor) [7,8], total laser time or energy [6] and even anesthetic technique [9]. However, the same data for ThuFLEP is not available. In a series of studies from Dmitry Enikeev et al. it was reported about UI with different ranges depending on type [3,10,11], but no one has investigated the risk factors of UI following ThuFLEP.

Thus, in this study we aimed to evaluate the predictive clinical and operative risk factors for UI rates following ThuFLEP.

## Materials and methods

### Study design

After obtaining Institutional Review Board approval, we conducted a retrospective cohort review of a prospectively maintained database for patients who underwent ThuFLEP between 2021 and 2023 in our university clinic performed by a single surgeon with high experience (more than 3000 endoscopic enucleation). Patients with a history of previous transurethral surgery before enucleation, urethral stricture, prostate cancer, or neurogenic bladder were excluded.

The data regarding the patient’s demographics, comorbidities, preoperative and perioperative features, including continence status were collected. From preoperative features International Prostate Symptom Scores (IPSS), quality of life score (QoL), maximum urination rate (Qmax), prostate specific antigen (PSA), American Society of Anesthesiologists score (ASA) were assessed. Prostate volume and postvoid residual (PVR) were assessed preoperatively using transrectal ultrasound and transabdominal ultrasound of the urinary bladder respectively. The operative time required for enucleation and for morcellation were separately identified.

Postoperative outcomes regarding UI and its type during three follow-up intervals persistence (up to 1 months, from 1 to 3 months and after 3 months post-ThuFLEP) were collected. UI was defined as involuntary leakage of urine in accordance with the recommendations of the International Continence Society (7^th^ Edition) [12]. Both objective (bladder diary) and subjective (patient reported outcomes) were used for assessment of type and symptoms and bother of UI [12]. All patients were informed about possible complications, including UI, before ThuFLEP and were instructed about Kegel’s exercises for postoperative period. Instruction for Kegel’s exercises were given according to «Pelvic Floor Muscle (Kegel) Exercises for Males» from Memorial Sloan Kettering Cancer Center [13].

### ThuFLEP procedure

All operations were performed by one highly experienced surgeon. To perform the operation, we used a thulium fiber laser Fiberlase U3 (IRE Polus ltd, Fryazino, Russia) with operating modes of 1.5 J and 40 Hz 60 W. The fiber used is 550 microns. An irrigation resectoscope of the Iglessias type was used for fiber delivery, 27 SN. Morcellation was performed using a Piranha rotary morcellator (Richard Wolf, GmbH, Germany).

When performing the operation, three-lobe, two-lobe, and en block techniques were used, depending on the endoscopic anatomy of the prostate and its volume. The first stage was performed for all patients to enter the layer in the area of the seminal tubercle. When performing an early apical release, the mobilization of the lateral lobes occurred before 4 and 8 o’clock using incision of the mucosa around the sphincter, after which the dissection of the apical zone was performed. Subsequently, the operation techniques did not differ from the classical ones [14].

### Statistical analysis

The statistical analysis was performed using Statistica 12 (StatSoft, Inc., USA). The Shapiro – Wilk’s and Kolmogorov-Smirnov’s tests were used to assess the normality of continuous data. All data had non-normal distribution and was represented as median and interquartile range (IQR). The numeric data between groups were compared with Mann – Whitney U test. Categorical data was represented as number of patients (%). The categorical data between groups were compared using Pearson’s chi-square test, Pearson’s chi-square test with Yates’s continuity correction and one-tailed Fisher’s exact test.

Univariate and multivariable logistic regression analysis was used to determine factors independently associated with UI at different timepoints. The statistical significance was set at *p* < 0,05.

## Results

Out of total 578 patients underwent ThuFLEP, the study included 381 patients with full information about continence status. Median age was 67.0 years old (IQR 61.0–72.0) with median prostate volume 82.0 cm3 (IQR 50.0–115.0). Post-ThuFLEP UI was observed in 109 (28.6%) patients with duration less than 1 months in 46 (12.1%) cases, from 1 to 3 months in 57 (14.9%) cases and more than 3 months in 6 (1.6%) cases. The most common UI type was urge (11.8%), that was similar with stress (11.5%). But all UI was only short-time with more than 3 months only in 1.6% patients: 0.8% for stress UI, 0.3% for urge UI and 0.5% for mixed. Table 1 shows the distribution of different incontinence types and Kegel exercises performing.

**Table 1.** Type of incontinence in three different periods.

	Overall *n* = 109	Duration of urinary incontinence		
		< 1 months *n* = 46	1 to 3 months *n* = 57	> 3 months *n* = 6
Incontinence rate, n, (%)	109 (28.6%)	46 (12.1%)	57 (14.9%)	6 (1.6%)
Incontinence type, n, (%)				
Stress UI	44 (11.5%)	20 (5.2%)	21 (5.5%)	3 (0.8%)
Urge UI	45 (11.8%)	18 (4.7%)	26 (6.8%)	1 (0.3%)
Mixed UI	20 (5.2%)	8 (2.1%)	10 (2.6%)	2 (0.5%)
Kegel exercises, n, (%)	79 (72.5%)	39 (84.8%)	35 (62.5%)	5 (100.0%)

Next step we compared demographic, pre- and perioperative parameters between continent and incontinent patients overall. Results are shown in Table 2. Age, ASA score and comorbid diseases were comparable between groups, as well as most preoperative and perioperative parameters, included total prostate volume and total laser energy. Incontinent patients had significantly lower Qmax (*p*=0.017) and had different enucleation technique compare to continent patients. Continent patients more often underwent En-bloc ThuFLEP than incontinent patients (17.7% vs 5.5%, *p* < 0.001) (Table 2).

**Table 2.** Preoperative and intraoperative patient characteristics.

Parameters	Whole cohort (*n*=381)	Continent (*n*=272)	Incontinence (*n*=109)	р
Age, years, median (IQR)	67.0 (61.0–72.0)	67.0 (61.0–73.0)	67.0 (63.0–72.0)	0.240
ASA, score				0.745
1, n, (%)	24 (6.3%)	20 (7.4%)	4 (3.7%)	
2, n, (%)	313 (82.2%)	221 (81.3%)	92 (84.4%)	
3, n, (%)	29 (7.6%)	21 (7.7%)	8 (7.3%)	
4, n, (%)	9 (2.4%)	6 (2.2%)	3 (2.8%)	
5, n, (%)	6 (1.6%)	4 (1.5%)	2 (1.8%)	
Comorbid diseases				
Hypertension, n, (%)	256 (67.2%)	188 (69.1%)	68 (62.4%)	0.206*
Coronary heart disease, n, (%)	70 (18.4%)	51 (18.8%)	19 (17.4%)	0.764*
Diabetes mellitus, n, (%)	65 (17.1%)	51 (18,8)	14 (12.8%)	0.166*
Cerebrovascular diseases, n, (%)	44 (11.5%)	32 (11.8%)	12 (11.0%)	0.835*
**Preoperative parameters**				
Total prostate volume, cm3, median (IQR)	82.0 (50.0–115.0)	80.0 (60.0–109.2)	90.0 (60.0–120.0)	0.304
IPSS, score, median (IQR)	25.0 (21.0–30.0)	24.0 (21.0–30.0)	26.0 (21.0–32.0)	0.141
QoL, score, median (IQR)	5.0 (4.0–6.0)	5.0 (4.0–6.0)	5.0 (4.0–6.0)	0.745
Qmax, ml/sec, median (IQR)	9.2 (7.2–12.0)	9.6 (7.5–12.0)	8.3 (6.5–11.0)	**0.017**
PVR, ml, median (IQR)	75.0 (46.0–120.0)	72.0 (40.0–120.0)	85.0 (56.0–115.0)	0.290
PSA, ng/dL median (IQR)	4.0 (2.2–7.0)	4.0 (2.3–7.1)	4.0 (1.8–6.9)	0.352
Indwelling catheter, n, (%)	86 (22.6%)	67 (24.6%)	19 (17.4%)	0.129*
Duration of indwelling catheter				0.535***
Less than 6 months, n, (%)	64 (75.3%)	50 (75.8%)	14 (73.7%)	
6 months and more, n, (%)	21 (24.7%)	16 (24.2%)	5 (26.3%)	
**Intraoperative parameters**				
Total laser energy, kJ, median (IQR)	55.0 (42.0–75.3)	54.5 (40.3–74.1)	58.4 (43.2–80.2)	0.143
Early sphincter release, n, (%)	318 (83.5%)	230 (84.6%)	88 (80.7%)	0.364*
Enucleation technique				<0.001*
3-lobe, n, (%)	21 (5.5%)	8 (2.9%)	13 (11.9%)	
2-lobe, n (%)	306 (80.3%)	216 (79.4%)	90 (82.6%)	
En-bloc, n (%)	54 (14.2%)	48 (17.7%)	6 (5.5%)	
Operation time, min, median (IQR)	75.0 (60.0–100.0)	75.0 (60.0–100.0)	75.0 (55.0–100.0)	0.989
Enucleation time, min, median (IQR)	55.0 (40.0–75.0)	55.0 (40.0–70.0)	50.0 (40.0–80.0)	0.979
Morcellation time, min, median (IQR)	25.0 (17.0–30.0)	20.0 (15.0–30.0)	25.0 (20.0–35.0)	0.275
Anesthesia				0.342*
General, n, (%)	52 (13.6%)	40 (14.7%)	12 (11.0%)	
Spinal, n, (%)	329 (86.4%)	232 (85.3%)	97 (89.0%)	
Catheterization time, days, median (IQR)	2.0 (2.0–3.0)	2.0 (2.0–3.0)	2.0 (2.0–3.0)	0.758

* Pearson’s chi-square test with Yates’s continuity correction; **one-tailed Fisher’s exact test; without *Pearson’s chi-square test.

Table 3 shows univariate logistic regression analysis of risk factors for UI following ThuFLEP during 3 intervals: up to 1 months, from 1 to 3 months and after 3 months post-ThuFLEP. There are no preoperative variables associated with UI. Age. ASA score, comorbid diseases, total prostate volume, IPSS, QoL, Qmax, PSA, indwelling catheter and its duration were not significant predictors of UI at any follow-up.

**Table 3.** Univariate logistic regression analysis of risk factors for UI following ThuFLEP.

	Incontinence overall n=109		< 1 months n=46		1 to 3 months n=57		> 3 months n=6	
	OR (95% CI)	р	OR (95% CI)	р	OR (95% CI)	р	OR (95% CI)	р
Age, years	1.020 (0.992–1.049)	0.158	0.979 (0.933–1.027)	0.389	1.019 (0.971–1.069)	0.455	1.030 (0.934–1.136)	0.557
ASA, score	1.156 (0.810–1.651)	0.424	0.648 (0.313–1.339)	0.241	1.529 (0.746–3.137)	0.246	1.057 (0.285–3.922)	0.934
**Preoperative parameters**								
Total prostate volume, cm3	1.004 (0.999–1.008)	0.101	1.001 (0.994–1.008)	0.772	0.998 (0.991–1.005)	0.616	1.005 (0.994–1.017)	0.359
IPSS, score	1.038 (0.992–1.085)	0.106	1.045 (0.970–1.126)	0.244	0.953 (0.882–1.028)	0.213	1.031 (0.847–1.254)	0.762
Qmax, ml/sec	0.944 (0.880–1.012)	0.106	0.922 (0.823–1.033)	0.161	1.097 (0.974–1.236)	0.126	0.862 (0.588–1.264)	0.447
PVR, ml	1.000 (0.998–1.003)	0.901	1.002 (0.997–1.007)	0.461	0.998 (0.992–1.003)	0.377	1.004 (0.994–1.014)	0.473
Indwelling catheter	0.646 (0.367–1.138)	0.131	2.161 (0.791–5.900)	0.133	0.347 (0.127–1.106)	0.076	2.529 (0.429–14.929)	0.306
**Intraoperative parameters**								
Total laser energy, kJ	1.009 (1.001–1.018)	**0.036**	1.003 (0.990–1.016)	0.697	0.996 (0.983–1.010)	0.566	1.010 (0.986–1.035)	0.413
Early sphincter release vs standard	0.765 (0.429–1.365)	0.365	0.258 (0.080–0.828)	**0.023**	0.244 (0.075–0.792)	**0.019**	1.205 (0.133–10.894)	0.868
Enucleation technique (En-bloc vs 2-lobe or 3-lobe)	0.272 (0.113–0.656)	**0.004**	0.258 (0.029–2.285)	0.223	4.327 (0.487–38.423)	0.189	-	0.999
Operation time, min	1.002 (0.995–1.008)	0.659	0.992 (0.980–1.004)	0.178	1.008 (0.996–1.021)	0.167	0.999 (0.975–1.024)	0.942
Enucleation time, min	1.003 (0.994–1.013)	0.491	0.991 (0.976–1.006)	0.247	1.010 (0.994–1.026)	0.207	0.992 (0.957–1.029)	0.679
Morcellation time, min	1.011 (0.990–1.033)	0.287	0.964 (0.924–1.006)	0.091	1.036 (0.992–1.082)	0.107	1.023 (0.950–1.101)	0.553
Catheterization time, days	0.926 (0.758–1.131)	0.451	1.088 (0.758–1.562)	0.647	0.905 (0.627–1.305)	0.539	1.135 (0.563–2.287)	0.723

Among intraoperative characteristics, it was shown that higher total laser energy was associated with UI overall: OR=1.009; 95% CI=1.001–1.018; *p*=0.036. The same association was found for enucleation technique and UI overall: OR=0.272; 95% CI=0.113–0.656; *p*=0.004 in favor of En-bloc ThuFLEP. Early sphincter release was considered as protective factor for UI more than 1 month. So, early sphincter release decreases the probability of UI more than 1 months on 74,2% (OR=0.258; 95%CI=0.080–0.828; *p*=0.023) compare to UI less than 1 months. So early sphincter release is protective factor from longer UI. The same data were obtained for UI from 1 up to 3 months: early sphincter release during ThuFLEP were protective factor from UI more than 1 months. So, it was shown that without early sphincter release chance of UI from 1 up to 3 months increases on 75.6% (OR=0.244; 95%CI=0.075–0.792; *p*=0.019) compared to UI less than 1 month. Univariate analysis is detailed in Table 3.

A multivariate analysis was performed for 2 time points – up to 1 month and from 1- up to 3-months. Third timepoint have only few cases that are not enough for multivariate analysis. For overall incontinence rate only enucleation technique maintained statistical significance in favor En-bloc (OR=0.302; 95%CI=0.123–0.739; *p*=0.009). Early sphincter release was found as independent protective factor for early UI (up to 1 month). So, probability of UI more than 1 month increases on 74.3% without early sphincter release (OR=0.257; 95%CI=0.078–0.849; *p*=0.026) compare to UI less than 1 months. The same data were obtained for UI from 1 up to 3 months: early sphincter release during ThuFLEP were protective factor from UI more than 1 months. So, it was shown that without early sphincter release chance of UI from 1 up to 3 months increases on 75.6% (OR=0.244; 95%CI=0.073–0.818; *p*=0.022 compare to UI less than 1 month (Table 4).

**Table 4.** Multivariate logistic regression analysis of risk factors for UI following ThuFLEP.

	Incontinence overall n=109		< 1 month n=46		1 to 3 months n=57	
	OR (95% CI)	р	OR (95% CI)	р	OR (95% CI)	р
Age	1.020 (0.984–1.041)	0.413	1.015 (0.966–1.066)	0.562	1.011 (0.961–1.063)	0.675
Total prostate volume	1.000 (0.991–1.008)	0.931	1.003 (0.990–1.016)	0.656	1.003 (0.989–1.016)	0.711
Total laser energy	1.007 (0.991–1.023)	0.389	0.995 (0.970–1.020)	0.695	0.994 (0.969–1.020)	0.651
Early sphincter release vs standard	0.715 (0.395–1.294)	0.268	0.257 (0.078–0.849)	**0.026**	0.244 (0.073–0.818)	**0.022**
Enucleation technique (En-bloc/2-lobe or 3-lobe)	0.302 (0.123–0.739)	**0.009**	4.415 (0.484–40.259)	0.188	4.885 (0.535–44.615)	0.160

## Discussion

Postoperative UI, especially short-term, has become one of the most common complications after surgery for BPH, which becomes the main cause of postoperative anxiety and fear before surgery. The reported rate of UI after BPH enucleation is highly variable among different studies. According to systematic review and meta-analysis of randomized-controlled studies, published in 2022, overall stress UI was comparable after different types of endoscopic enucleation, as well as overall urge UI [15]. However, it was not reported about ThuFLEP.

According to Dmitry Enikeev et al.. (2019) short-term stress UI after ThuFLEP was noted in 6.7% patients, while at 3 months follow up – only in 4.4% [11]. Next publication from Dmitry Enikeev et al.. (2019) reported about 13.7% patients with short-term stress UI after ThuFLEP with 1.9% at 6- and 12-months [4]. One more study about comparison of HoLEP (*n*=77) and ThuFLEP (*n*=86) outcomes was conducted by Dmitry Enikeev et al. in 2022. Stress UI was found in 3.5% patients and 2.3% patients at 3- and 6 months respectively. For urge UI it was 1.2% and no patients at 3- and 6 months respectively. And mixed UI was reported in 1.2% at both 3- and 6-months [3].

The similar data about UI rates was found in our study, but long-term UI (more than 3 months) were found in less cases, than previously reported – only 1.6% with 0.8% for stress UI, 0.3% for urge UI and 0.5% for mixed UI. Such differences can be related to operation performance by one high experienced surgeon with more than 2000 endoscopic enucleation. It was also first study with thulium fiber laser Fiberlase U3, while before it was performed with Fiberlase U1. Firstly, for ThuFLEP we assessed risk factors for postoperative de novo UI with no the same data for these operation in literature.

Latest research reported about post-HoLEP UI risk factors as preoperative UI, obesity and large prostate volume [16], that was also confirmed earlier by Laura B Cornwell et al.. (2019) [17]. Xuanhao Li et al.. (2021) developed a nomogram for predicting early stress UI following different types endoscopic enucleation of the prostate [18]. According to multivariable logistic regression analysis showed that independent predictors of stress UI were older age, higher body mass index, longer duration of LUTS, and higher prostate volume [18]. The same study for HoLEP was also conducted by Vianney Houssin in 2021. In multivariate regression analysis increasing age, elevated body mass index, preoperative urinary drainage, increasing prostate volume and experienced surgeon were significantly associated with UI as well at 3- as 6-months [19]. Nevertheless, it was not reported about intra- and perioperative risk factors, including operation technique and total laser energy.

In similar study for HoLEP was reported that preoperative erectile dysfunction, higher delivered energy, higher enucleated prostate weight and total intraoperative time were statistically associated with the occurrence of stress UI postoperatively [20]. Anesthetic Technique (Spinal vs General Anesthesia) during HoLEP was not associated with UI [9], that was also confirmed in our study.

A systemic review and meta-analysis for incidence and risk factors for postoperative UI after various prostate enucleation procedures was published in 2022 [6]. The analysis includes 5 subgroups based on energy source: holmium laser, thulium laser (also include thulium fiber laser), greenlight laser, electrocautery and simple prostatectomy. It was shown that overall incidence of UI during 1–3 months post-HoLEP was 15%, during 3–6 months − 4% and after 6 months − 4%. None of the articles with thulium or thulium fiber laser using reported overall UI. They all specifically classified UI into stress or urge with separate analysis. After predictor analysis it was found that HoLEP is associated with a higher incidence of UI. It was also found that median age, surgery time and laser time appeared to be correlated with higher rates of UI at each timepoint. Finally, enucleation technique (en-bloc vs two/three-lobes), early sphincter release or prostate volume had any statistically significant impact on the incidence of postoperative UI. However, separate analysis for ThuFLEP was not conducted [6]. There was only 1 ThuFLEP article with only 90 patients [6,11].

In our study we conducted a full complex analysis for only ThuFLEP risk factors of de novo postoperative UI rates. According to univariate logistic regression analysis it was shown that higher total laser energy was associated with UI overall, while En-bloc ThuFLEP was protective factor for UI overall. On multivariable analysis En-bloc ThuFLEP was more powerful protective factor for UI than total laser energy. Early sphincter release was also found as protective factor for UI less than 1 months, that was proved before for bipolar enucleation [21]. No incontinence were found at 3 months follow-up compare to 3.7% for bipolar enucleation without protection of the external urethral sphincter in the beginning of surgery [21]. Potential advantages of early apical release with circumferential mucosal incision proximal to the sphincter ring are as follows: better integrity of the mucosa surrounding the external sphincter [22], less overstretch the sphincter when cutting the mucosal flap [23]. This allows to prevent external sphincter injury and can lead to potential reduction of post-operative SUI after surgery [24].

According to summary paper on the 2023 European Association of Urology Guidelines the choice of surgical technique depends on patient’s characteristics, expectations, and preferences, surgeon’s expertise and availability of modalities [25]. However, the surgeon needs to remember that some clinical baseline and perioperative data are considered to predict the probability of developing UI after BPH surgery. Preoperative information of the patients on the risk of urinary incontinence is essential.

Limitations of the current study include its origin from a single center with high experience with ThuFLEP, exclusion of a number of patients due to incomplete follow up with probable different UI rates and its retrospective nature, however all the patients’ data were prospectively maintained.

## Conclusion

Short-term UI up to 3 months following ThuFLEP is associated with early sphincter release as protective factor, while overall UI rates are related with enucleation technique (preferable En-bloc) and total laser energy (preferable lower energy). However, if surgeon use En-bloc ThuFLEP it is already not necessary to control energy because En-bloc is more powerful favorable factor for UI after ThuFLEP. The surgeon should take all this risk factors into account before choosing the technique of BPH surgical treatment.
